# Catastrophic costs potentially averted by tuberculosis control in India and South Africa: a modelling study

**DOI:** 10.1016/S2214-109X(17)30341-8

**Published:** 2017-10-09

**Authors:** Stéphane Verguet, Carlos Riumallo-Herl, Gabriela B Gomez, Nicolas A Menzies, Rein M G J Houben, Tom Sumner, Marek Lalli, Richard G White, Joshua A Salomon, Ted Cohen, Nicola Foster, Susmita Chatterjee, Sedona Sweeney, Inés Garcia Baena, Knut Lönnroth, Diana E Weil, Anna Vassall

**Affiliations:** a Department of Global Health and Population, Harvard T H Chan School of Public Health, Boston, MA, USA; b Department of Global Health, Amsterdam Institute for Global Health and Development, Academic Medical Centre, University of Amsterdam, Amsterdam, Netherlands; c TB Modelling Group, TB Centre, London School of Hygiene and Tropical Medicine, London, UK; d Department of Infectious Disease Epidemiology, London School of Hygiene and Tropical Medicine, London, UK; e Department of Global Health and Development, London School of Hygiene and Tropical Medicine, London, UK; f Department of Epidemiology of Microbial Diseases, Yale School of Public Health, New Haven, CT, USA; g Health Economics Unit, School of Public Health and Family Medicine, University of Cape Town, Cape Town, South Africa; h Public Health Foundation of India, New Delhi, India; i Global TB Programme, WHO, Geneva, Switzerland; j Department of Public Health Science, Karolinska Institutet, Stockholm, Sweden

## Abstract

**Background:**

The economic burden on households affected by tuberculosis through costs to patients can be catastrophic. WHO's End TB Strategy recognises and aims to eliminate these potentially devastating economic effects. We assessed whether aggressive expansion of tuberculosis services might reduce catastrophic costs.

**Methods:**

We estimated the reduction in tuberculosis-related catastrophic costs with an aggressive expansion of tuberculosis services in India and South Africa from 2016 to 2035, in line with the End TB Strategy. Using modelled incidence and mortality for tuberculosis and patient-incurred cost estimates, we investigated three intervention scenarios: improved treatment of drug-sensitive tuberculosis; improved treatment of multidrug-resistant tuberculosis; and expansion of access to tuberculosis care through intensified case finding (South Africa only). We defined tuberculosis-related catastrophic costs as the sum of direct medical, direct non-medical, and indirect costs to patients exceeding 20% of total annual household income. Intervention effects were quantified as changes in the number of households incurring catastrophic costs and were assessed by quintiles of household income.

**Findings:**

In India and South Africa, improvements in treatment for drug-sensitive and multidrug-resistant tuberculosis could reduce the number of households incurring tuberculosis-related catastrophic costs by 6–19%. The benefits would be greatest for the poorest households. In South Africa, expanded access to care could decrease household tuberculosis-related catastrophic costs by 5–20%, but gains would be seen largely after 5–10 years.

**Interpretation:**

Aggressive expansion of tuberculosis services in India and South Africa could lessen, although not eliminate, the catastrophic financial burden on affected households.

**Funding:**

Bill & Melinda Gates Foundation.

## Introduction

In May, 2014, the World Health Assembly ratified the End TB Strategy, which set aspirational worldwide objectives for tuberculosis control: reductions of 50% in global tuberculosis incidence and 75% in tuberculosis mortality by 2025, and of 90% and 95%, respectively, by 2035.^1^ National policy makers are now deciding which interventions to implement to achieve these targets. The TB Modelling and Analysis Consortium assessed in its TB Targets exercise^2^ how expanding control by an ambitious but feasible degree might affect the targets being achieved in China, India, and South Africa.

In China, sustained improvements in tuberculosis interventions led to a 70% decline in prevalence from 1990 to 2010,^3^, ^4^ and by 2015, tuberculosis incidence had reached 70 cases per 100 000 population.^3^ By contrast, in South Africa, where the tuberculosis epidemic has been greatly affected by HIV dynamics,^5^, ^6^ declines in tuberculosis incidence have been seen since only around 2010, and in 2015 incidence was 830 cases per 100 000.^3^ India has had relatively stable incidence of 200–300 cases per 100 000 population for the past 15 years, but faces challenges associated with a large and heterogeneous private sector that is used by many patients for diagnosis and care. By expanding government subsidies, India has attempted to improve the quality of tuberculosis care provided in the private sector.^7^

The TB Targets exercise was a modelling study done to assess the hypothetical effects of specific TB interventions and showed that the 2025 targets of the End TB Strategy would not be achieved by implementing any one intervention.^2^ In India and China, targets might only be met with aggressive implementation of a comprehensive set of currently available interventions and if new and improved technologies become available.^2^ Improvements need to be made in access to high-quality tuberculosis care, diagnosis, multidrug resistance (eg, by replacing smear microscopy with molecular diagnostic techniques), adherence and treatment success rates, active case finding in the general population, and use of isoniazid preventive therapy for people taking antiretroviral therapy.^2^ A concurrent economic evaluation found that such expansion was likely to be cost-effective and would avert large numbers of deaths per incremental tuberculosis budget dollar, but might need substantial increases in funding at the time of implementation,^8^ which in India and South Africa, would lead to annual tuberculosis service costs more than doubling and rising to 3–4% of current public health financing.

**Research in context**

**Evidence before this study**

WHO's End TB Strategy proposes aggressive action to reduce tuberculosis incidence and mortality. Associated with those goals will be reductions in related out-of-pocket costs and impoverishment, which can be catastrophic when they place excessive economic burden on households. Although the economic burden of tuberculosis on households is known to be high, there is little evidence reported on the effects of financial-risk protection strategies in high-burden settings, such as India and South Africa. The TB Modelling and Analysis Consortium highlighted two reports on the End TB Strategy which suggested through estimation of costs incurred by patients in high-burden settings that substantial improvements in tuberculosis control could be cost-effective.

**Added value of this study**

We used models to estimate reductions in tuberculosis-related catastrophic costs with aggressive expansion of tuberculosis services in India and South Africa from 2016 to 2035. We quantified intervention effects as changes in the number of households incurring catastrophic costs, which were defined as the sum of direct medical and non-medical costs and indirect costs being more than 20% of the total household income. We estimated that improved care for drug-sensitive and multidrug-resistant tuberculosis would lead to reductions of up to 19% in India and South Africa, and found that benefits would be greatest in the poorest households. In South Africa, the expansion of access to care through intensified case finding could decrease tuberculosis-related household catastrophic costs by up to 20%.

**Implications of all the available evidence**

Aggressive expansion of tuberculosis services in India and South Africa could alleviate the financial burden on many patients. Nevertheless, improved social protection for people with tuberculosis will still be needed to meet the End TB Strategy target of eliminating catastrophic costs. Our results support efforts to scale up tuberculosis services and moving towards universal health coverage.

The TB Targets exercise suggested that expanding tuberculosis control would substantially reduce costs incurred by patients^8^ and thereby also diminish tuberculosis-related household impoverishment. This outcome would be important given the WHO's aim of universal health coverage and the fact that out-of-pocket medical costs are a leading cause of impoverishment in many low-income and middle-income countries.^9^ Survey evidence also suggests that in such settings tuberculosis leads to substantial losses in wages and work productivity for the affected individuals and their households.^10^

One of the three goals of the End TB Strategy is that no patient or their household should face catastrophic costs because of tuberculosis.^11^, ^12^ This goal is consistent with WHO having made protection from financial risks and prevention of medical impoverishment key elements of health-system performance^13^ and included financial protection in universal health coverage.^14^ Given the association between tuberculosis and poverty, and the recommendation to monitor related catastrophic costs,^11^, ^12^ it is essential to understand how different interventions might prevent impoverishment. Traditional cost-effectiveness analysis may be complemented by extended cost-effectiveness analysis (ECEA), which estimates the distributional and financial protection benefits to be gained by resource allocation^15^, ^16^ and, hence, when combined with modeling, can explore the financial protection impact of different tuberculosis interventions.

We developed a mathematical model to assess whether tuberculosis-related household catastrophic costs could be reduced by expansion of tuberculosis interventions in India and South Africa, building on the TB Targets exercise^2^, ^8^ and consistent with ECEA. We compared a subset of interventions highlighted by the TB Targets exercise with a base case derived from the standards of tuberculosis care in these countries at the start of the study.

## Methods

### Study design

We did a modelling study to assess effects of different interventions on catastrophic costs related to tuberculosis in the period 2016–35. We selected India and South Africa for this study because they are both major contributors to the global tuberculosis burden but differ in HIV and tuberculosis epidemiology, approaches to tuberculosis control, mix of public and private health-care provisions, and the availability of data on costs incurred by patients; although the main TB Targets analysis also included China, we only selected India and South Africa because of the criteria specified. India has the largest national tuberculosis burden worldwide and a large private sector providing care to about half of affected individuals.^17^ South Africa has the largest tuberculosis burden per person worldwide, and HIV and tuberculosis are closely linked in this country.^6^

### Intervention scenarios

In each country, we consulted extensively with representatives from national tuberculosis programmes to decide on the activities that would be most relevant to assess with existing tools. We selected interventions with clear information on implementation and coverage, and with detailed future scenarios based on a framework of tuberculosis programme activities (table 1, appendix p 2). We assessed the effects of improvement in treatment quality for drug-sensitive and multidrug-resistant tuberculosis in South Africa and India. In India, there are many treatment providers of widely varying qualities,^17^, ^18^, ^19^ and, therefore, the treatment interventions allowed exploration of achievements if increased proportions of individuals with tuberculosis could access higher-quality care in this country (similar to optimum public sector care). We also assessed expansion of access to care through intensified tuberculosis screening at primary-care clinics in South Africa. In the intervention scenarios, high coverage was reached by 2025 and remained constant for 10 years over 2025–35. The models also considered local policy constraints and system capacities.^2^, ^8^

**Table 1:** Intervention scenarios for India and South Africa^8^

	**Activities**	**Programme targets**	**Mechanism of action for health effects**
**India**			
Improving treatment quality	Improved private-sector quality through provider training, supervision, regulation, and subsidies; retention of patients in care by incentives, nutritional support, and link to social welfare programmes	Initial decrease in patients stopping treatment from 10% to 5% by 2015 for DS-TB and from 11% to 5% by 2020 for MDR-TB; treatment success measured as increases in adherence from 75% to 85% and from 48% to 67%, respectively	Improved retention of patients receiving care leading to increased cure rates
**South Africa**			
Expanding access to care	Outreach clinics to underserved areas and symptom screening in primary care	Decrease population without access to care from 5% to zero by 2022	Reduced duration of infectiousness and mortality risks by improved case detection
Improving treatment quality	Mobile health care, follow-up of patients in the community, counselling on adherence to treatment, and improved MDR-TB staffing	Initial decreases in patients stopping treatment from 17% to 5% by 2021 for DS-TB and from 30% to 15% by 2021 for MDR-TB; treatment success measured as increases in adherence from 76% to 85% and from 52% to 67%, respectively	Improved retention of patients receiving care leading to increased cure rates

DS-TB=drug-sensitive tuberculosis. MDR-TB=multidrug-resistant tuberculosis.

In India, we took improvement in quality of treatment to mean that treatment adherence would increase from 75% to 85% for drug-sensitive tuberculosis and from 48% to 67% for multidrug-resistant tuberculosis. These changes would be achieved in both intervention scenarios through improved quality of private-sector treatment by provider training, supervision, regulation, and subsidies, and provision of patient-retention incentives, nutritional support, and linkage to social welfare programmes. In South Africa, we took improvement in quality of treatment to mean that treatment adherence would increase from 76% to 85% for drug-sensitive tuberculosis and from 52% to 67% for multidrug-resistant tuberculosis. These changes would be achieved in both intervention scenarios through provision of mobile health care and follow-up in the community, offering patients adherence counselling and psychosocial support, and tracing of patients to avert care dropout. Additional features to achieve improved treatment of multidrug-resistant tuberculosis would be increased staffing and decentralisation of the electronic register. We took expansion of access to care in South Africa to mean that all individuals seeking care would be screened for symptoms of tuberculosis.

We compared each intervention scenario with a base case, which assumed that coverage and treatment success rates at the start of the study would be maintained at a constant for the period 2016–35. The baseline trends in tuberculosis incidence and mortality projected in the base case were inferred from model calibration with 2012 survey data and past trends from 2000–15.^2^ The base case included country standards of care derived from the FIND study in India^20^ and the XTEND study in South Africa.^21^, ^22^

### Calculation of catastrophic costs

We defined catastrophic costs as the sum of direct medical costs (eg, treatment costs), direct non-medical costs (eg, transport and food during visits), and indirect costs (eg, loss of earnings) to the patient exceeding 20% of total annual household income.^23^, ^24^ This definition followed that in WHO's protocol to estimate the proportion of households experiencing tuberculosis-related catastrophic costs.^24^ A simplifying assumption was made that each household could have a maximum of one case of tuberculosis.

We calculated household catastrophic costs for each intervention scenario and the base case. Indirect costs were estimated for the time before diagnosis of tuberculosis when patients were not taking treatment and, therefore, reductions due to interventions were quantified as estimated reductions in the time from onset of tuberculosis symptoms to diagnosis (table 2).

**Table 2:** Parameters used to estimate catastrophic costs averted

		**India**	**South Africa**	**References**
**Epidemiology**				
Cumulative numbers (2016–35) in base case in Harvard model and TIME model				
	Treated DS-TB cases	49 785 000, 32 877 000	6 211 000, 5 342 000	2
	Treated MDR-TB cases	851 000, 1 258 000	160 000, 318 000	2
	Tuberculosis-related deaths	6 547 000, 8 210 000	1 316 000, 1 345 000	2
Estimated relative risk of tuberculosis, from poorest to richest quintile		1·00, 0·66, 0·50, 0·28, 0·18	1·00, 0·66, 0·57,0·47, 0·17	25, 26
Estimated relative ratio of health-care use, from poorest to richest quintile		0·18, 0·39,0·59, 0·80, 1·00	0·67, 0·75,0·83, 0·92, 1·00	27, 28
**Direct medical costs to patients**				
Monthly costs (US$)				
	DS-TB care (base case)	61	38	8, 10, 29, 30
	MDR-TB care (base case)	61	123	8, 29, 30, 31
	Improved DS-TB care	48	38	8, 10, 29, 30
	Improved MDR-TB care	48	123	8, 29, 30, 31
Fixed costs (per visit)*		42	N/A	8, 10, 29, 30
**Direct non-medical and indirect costs to patients**				
Average time from onset to diagnosis in base case in Harvard model and TIME model (months)		11·9, 20·4	9·7, 9·9	2
Average time from onset to diagnosis with expansion of access to care in Harvard model and TIME model (months)		N/A	6·6, 6·9	2
Diagnosis cost per month ($)†		60	66	8, 10, 29, 30
Funeral costs ($)		300	1850	32, 33
Annual income per capita ($)		1600	6890	34
Gini index‡		0·33	0·65	34
Distribution of annual income per capita by income quintile ($)				
	1	<750	<2760	··
	2	750–1170	2760–4530	··
	3	1170–1640	4530–6580	··
	4	1640–2320	6580–9630	··
	5	>2320	>9630	··

DS-TB care implies 6 months of treatment and MDR-TB care implies 24 months of treatment. In the base case, all coverage levels and treatment success rates at the start of the study were assumed to be maintained at a constant for the period 2016–35. Costs are expressed in 2014 US$. TIME=TB Impact Model and Estimates. DS-TB=drug-sensitive tuberculosis. MDR-TB=multidrug-resistant tuberculosis. N/A=not applicable.

* Include per-visit costs to physicians for diagnosis of tuberculosis.

† Includes indirect social care and transport costs per month until tuberculosis is diagnosed. Reduction in indirect cost is then valued through the reduction of time to tuberculosis diagnosis after onset of interventions.

‡ Measure of inequality of income distribution.

### Modelling

Modelling involved four steps for each country. Step 1 involved collecting the number of tuberculosis cases and deaths for each intervention and year. In line with the TB Targets exercise, we used two dynamic deterministic compartmental models of tuberculosis transmission, the Harvard model developed by Menzies and colleagues^35^ and the model of the TB Impact Model and Estimates group (appendix p 1).^36^ Both models use the following factors to stratify populations: drug-sensitive or multidrug-resistant tuberculosis, treatment history for tuberculosis, HIV status, receiving antiretroviral therapy, and CD4 cell count. The Harvard model followed one age group (>15 years) and used Bayesian calibration, whereas the TB Impact Model and Estimates model tracked the whole population and used manual calibration.^2^ We used the following model outputs to estimate patient-incurred costs throughout the period 2016–35: the number of tuberculosis cases screened and diagnosed; the numbers of patients with tuberculosis who received treatment for drug-sensitive tuberculosis or for multidrug-resistant tuberculosis; time from onset of tuberculosis symptoms to starting treatment; and the number of tuberculosis-related deaths (table 2). The estimated numbers of tuberculosis cases screened, diagnosed, and treated for drug-sensitive or multidrug-resistant tuberculosis, as estimated by the models for the base case and the intervention scenarios, were distributed across income quintiles by an income distribution analysis. Data for tuberculosis prevalence^25^, ^26^ and health-care use^27^, ^28^ were obtained from the literature. We used case-fatality ratios to allocate tuberculosis-related deaths between people who were treated and those who remained untreated.^37^

In step 2, we used the estimates of direct and indirect costs incurred by patients (table 2), which were based on an analysis of surveys by Menzies and colleagues (appendix pp 3–7),^8^ to assess the effects of each intervention. These estimates provided the unit cost per model output from the patient's perspective and estimated a mean cost per tuberculosis-affected household. Indirect costs compared with average income were varied by income quintile (–20%, −10%, 0, 10%, 20% in quintiles 1 to 5, respectively), and we included costs for tuberculosis-related deaths (eg, funeral costs^32^, ^33^).

In step 3, we assigned an annual income for each tuberculosis-affected household, based on the income distribution defined for each tuberculosis case in step 1, drawn from a simulated γ distribution with parameters based on the country's gross domestic product per capita and Gini index (table 2).^34^, ^38^, ^39^ We then determined the size of each household with a Poisson model, with the average size taken from each country's national census (four per household in South Africa and five in India).^40^, ^41^ This approach allowed us to define household annual income in each quintile (table 2) and thus to which income quintile each case of tuberculosis should be assigned.

In step 4, we calculated the number of households incurring catastrophic costs per year and cumulatively in 2016–35, by income quintile. We combined the estimated household income for each quintile with the patient-incurred costs estimated in step 2 (table 2). To obtain the total number of cases of catastrophic costs averted by each intervention scenario, we subtracted the number of households incurring catastrophic costs in the base case from the number in the intervention scenario (appendix pp 8–9).

### Further sensitivity and scenario analyses

We did three univariate sensitivity analyses to assess the effects of key factors, varying the following factors by 50% above and below baseline values: direct costs, funeral costs, and health-care use (appendix pp 24–35). Furthermore, we ran a probabilistic sensitivity analysis with Monte Carlo simulations (n=100 000 trials), where time to diagnosis, health-care use, income, and costs to patients were varied simultaneously with truncated normal distributions, with input means extracted from the model outputs and 20% of those means used to calculate SDs. From these data we extrapolated 2·5 and 97·5 percentiles to determine 95% uncertainty ranges.

We did four scenario analyses. First, we varied the burden distribution and assumed that tuberculosis cases were equally distributed across income quintiles (ie, removing the assumption that tuberculosis would occur more in the poor). Second, we varied the distribution of health-care use and assumed equal access to care across each quintile (ie, removing the assumption that the richer patients would seek care more than the poorer patients). Third, we modified the patient-incurred costs by removing costs arising from tuberculosis-related deaths and assuming equal indirect costs across quintiles. Fourth, we changed the catastrophic costs metric by changing the threshold from 20% to 10% or 40% of total household income^42^, ^43^, ^44^ or by using a different threshold for each quintile (eg, 20%, 25%, 30%, 35%, and 40%). All costs were expressed in 2014 US$. All analyses were done with RStudio (version 1.0.136).

### Role of the funding source

The funder had no role in the study design, data collection, data analysis, data interpretation, or writing of the report. The corresponding author had full access to all the data in the study and had final responsibility for the decision to submit for publication.

## Results

The estimated numbers of households that incurred tuberculosis-related catastrophic costs in 2016–35, were around 20 million to 22 million in India and 1·1 million to 1·2 million in South Africa (Table 3, Table 4). We found a substantially higher risk of catastrophic costs being incurred in the bottom 40% of households in each country, among which the prevalence of tuberculosis was highest. In India, 30–40% of all catastrophic costs were estimated to be in the bottom income quintile, and in South Africa the proportion was about 80%.

**Table 3:** Estimated numbers of households (in thousands) incurring tuberculosis-related catastrophic costs in India

	**Total number of households (95% UR)**	Number of households (95% UR) per income quintile*				
		1	2	3	4	5
**Harvard model**						
Base case	20 596 (16 848–24 278)	8785 (8276–9203)	5955 (4334–7394)	4199 (3024–5449)	1332 (920–1874)	326 (197–512)
Improvement in DS-TB care	19 544 (15 976–23 049)	8357 (7864–8766)	5638 (4104–7006)	3979 (2864–5168)	1261 (871–1775)	309 (187–486)
Improvement in MDR-TB care	20 475 (16 753–24 130)	8739 (8232–9155)	5917 (4309–7349)	4171 (3005–5413)	1324 (915–1860)	324 (196–509)
**TIME model**						
Base case	21 926 (18 864–24 629)	6757 (6442–7106)	7101 (6370–7571)	5383 (3854–6685)	2036 (1425–2740)	649 (493–826)
Improvement in DS-TB care	20 547 (17 690–23 079)	6381 (6065–6732)	6635 (5950–7074)	5027 (3602–6243)	1898 (1329–2556)	605 (459–771)
Improvement in MDR-TB care	21 790 (18 742–24 485)	6696 (6378–7049)	7059 (6327–7526)	5360 (3839–6659)	2028 (1419–2731)	647 (491–824)

Catastrophic costs are defined as the sum of costs exceeding 20% of total household income. In the base case, all coverage levels and treatment success rates at the start of the study were assumed to be maintained at a constant for the period 2016–35. UR=uncertainty range. DS-TB=drug-sensitive tuberculosis. MDR-TB=multidrug-resistant tuberculosis. TIME=TB Impact Model and Estimates.

* From poorest (quintile 1) to richest (quintile 5).

**Table 4:** Estimated number of households (in thousands) incurring tuberculosis-related catastrophic costs in South Africa

	**Total number of households (95% UR)**	Number of households (95% UR) per income quintile*				
		1	2	3	4	5
**Harvard model**						
Base case	1184 (1031–1348)	925 (805–1049)	195 (163–231)	59 (42–78)	5 (0–11)	0 (0–2)
Expansion of access to care	1123 (972–1279)	882 (769–999)	199 (165–235)	38 (22–57)	3 (0–7)	0 (0–2)
Improvement in DS-TB care	964 (836–1103)	757 (657–861)	158 (129–190)	45 (30–61)	4 (0–9)	0 (0–2)
Improvement in MDR-TB care	965 (837–1105)	759 (658–865)	157 (129–190)	44 (30–60)	4 (0–9)	0 (0–2)
**TIME model**						
Base case	1243 (1085–1396)	984 (864–1096)	172 (141–205)	81 (58–103)	7 (1–14)	0 (0–1)
Expansion of access to care	999 (874–1130)	802 (709–898)	143 (113–173)	49 (33–68)	5 (0–12)	0 (0–1)
Improvement in DS-TB care	1152 (1005–1297)	915 (803–1021)	159 (129–190)	72 (52–93)	6 (0–14)	0 (0–1)
Improvement in MDR-TB care	1171 (1016–1320)	934 (816–1044)	157 (127–189)	73 (52–94)	6 (0–14)	0 (0–1)

Catastrophic costs are defined as the sum of costs exceeding 20% of total household income. In the base case, all coverage levels and treatment success rates at the start of the study were assumed to be maintained at a constant for the period 2016–35. UR=uncertainty range. DS-TB=drug-sensitive tuberculosis. MDR-TB=multidrug-resistant tuberculosis. TIME=TB Impact Model and Estimates.

* From poorest (quintile 1) to richest (quintile 5).

**Figure 1:**
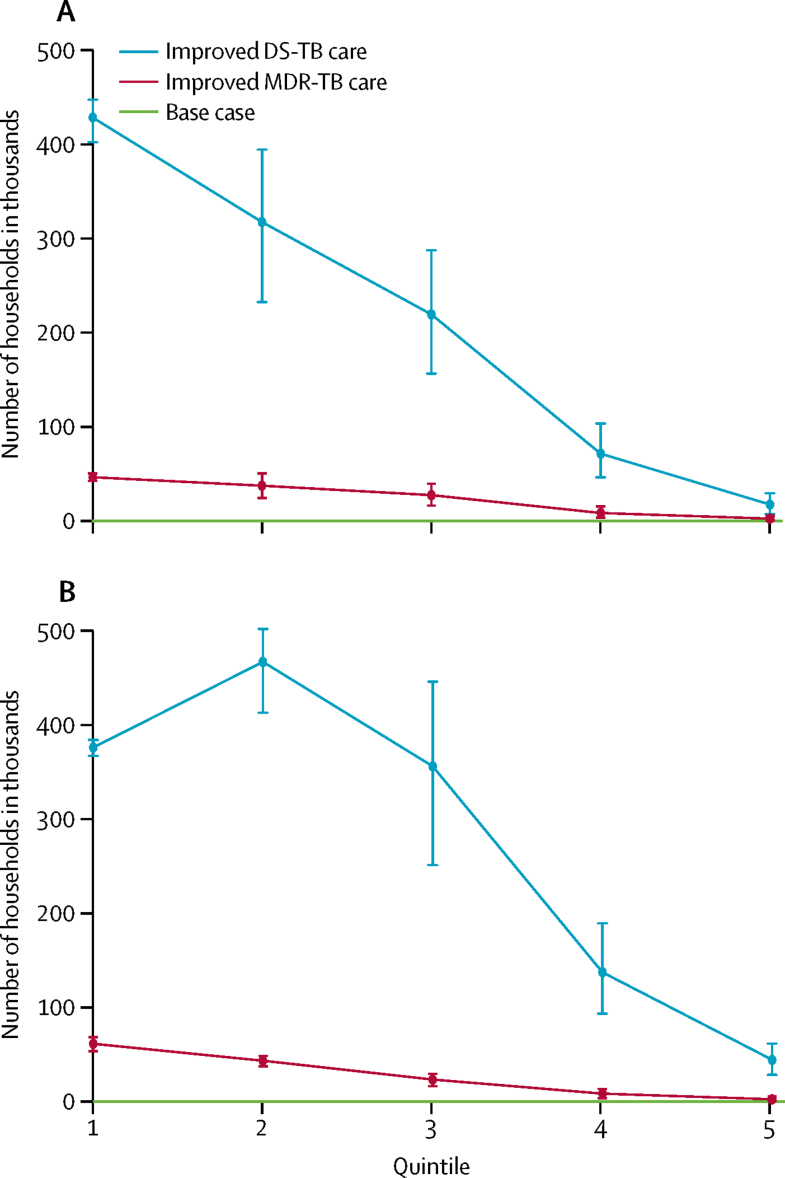
Number of households in India with catastrophic costs averted by improved tuberculosis care, compared with the base case, by income quintile (A) Harvard model. (B) TB Impact Model and Estimates model. The base case covers the period 2016–35, during which all coverage levels and treatment success rates were assumed to be maintained at a constant. Numbers of households are shown with 95% uncertainty ranges. Quintiles range from poorest (quintile 1) to richest households (quintile 5). DS-TB=drug-sensitive tuberculosis. MDR-TB=multidrug-resistant tuberculosis.

**Figure 2:**
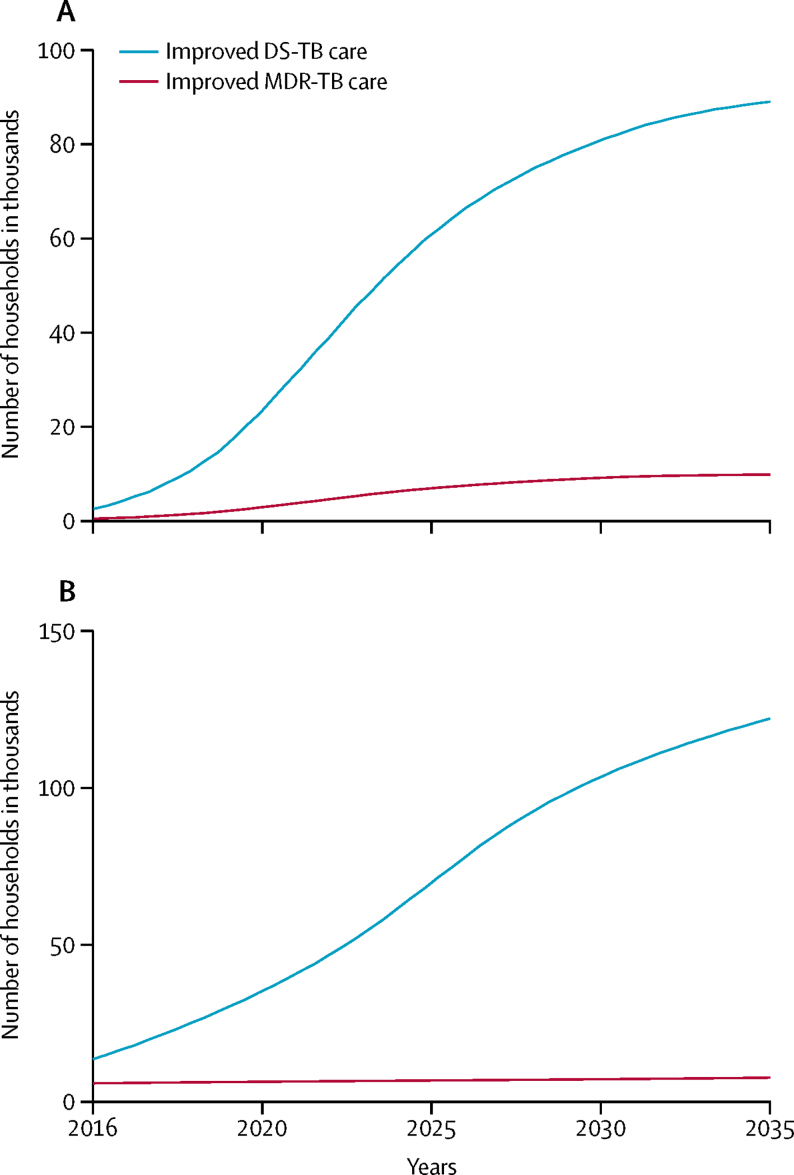
Number of households in India per year with catastrophic costs averted by improved tuberculosis care over the period 2016–35 (A) Harvard model. (B) TB Impact Model and Estimates model. DS-TB=drug-sensitive tuberculosis. MDR-TB=multidrug-resistant tuberculosis.

In India, more catastrophic costs would be averted by improvements in drug-sensitive tuberculosis care than by improvements in multidrug-resistant tuberculosis care (figure 1), and the difference between these two interventions would increase over time (figure 2). Over 2016–35, improvements in care for drug-sensitive tuberculosis would avert about 1·1 million to 1·4 million cases of household catastrophic costs (table 3), which was about 5–6% of all cases in the base case. Improvements in care of multidrug-resistant tuberculosis would avert about 120 000–140 000 cases or up to 1% of all cases incurred in the base case (table 3). These cost reductions would occur because of the provision of financial incentives to patients in the intervention and the prevention of additional tuberculosis transmission and infections.^2^, ^8^ The benefits of these two interventions would be greatest in the lowest two income quintiles (poorest 40% of households), where 60–70% of cases of catastrophic costs would be averted by improvements in care of drug-sensitive tuberculosis and 70–80% would be averted by improvements in care for multidrug-resistant tuberculosis. These reductions would be due to decreasing tuberculosis incidence, increasing health-care use, and decreasing likelihood of catastrophic costs with increasing income.

**Figure 3:**
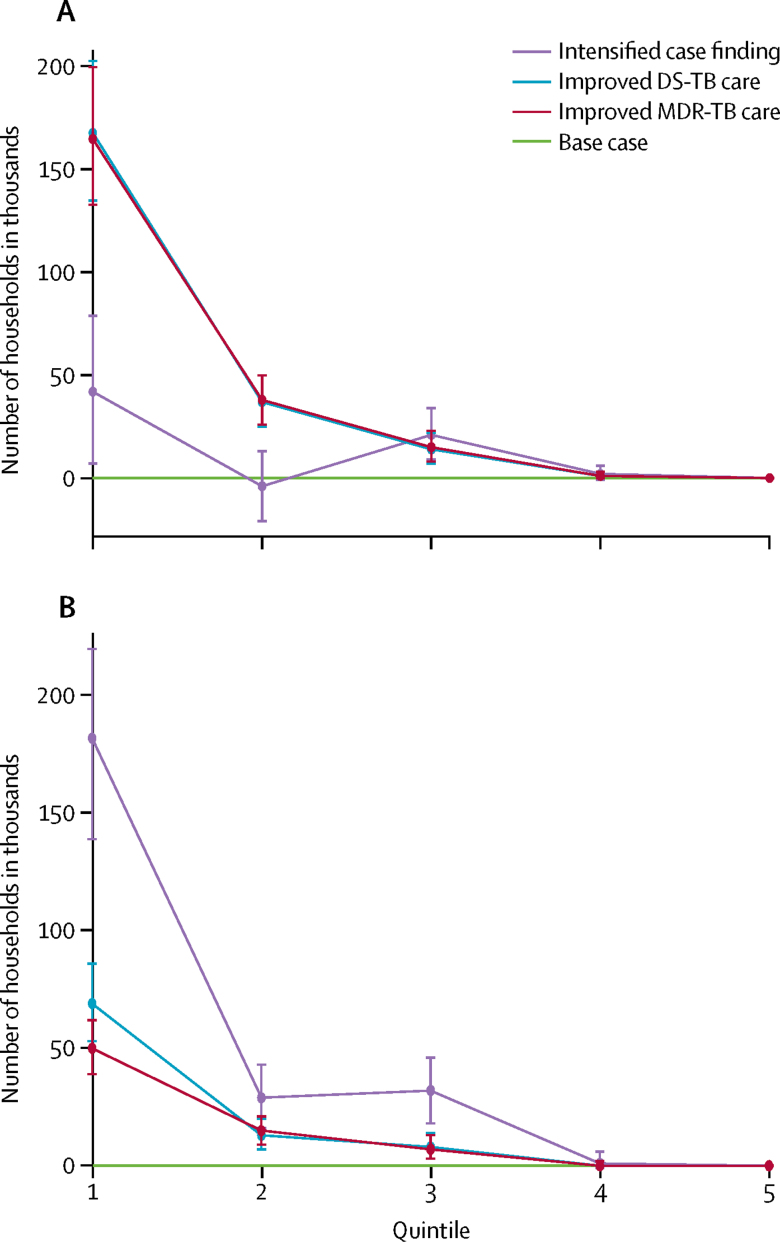
Number of households in South Africa with catastrophic costs averted by intensified case finding and improved tuberculosis care, compared with the base case, by income quintile (A) Harvard model. (B) TB Impact Model and Estimates model. The base case covers the period 2016–35, during which all coverage levels and treatment success rates were assumed to remain at a constant. Numbers of households are shown with 95% uncertainty ranges. Quintiles range from poorest (quintile 1) to richest households (quintile 5). DS-TB=drug-sensitive tuberculosis. MDR-TB=multidrug-resistant tuberculosis.

**Figure 4:**
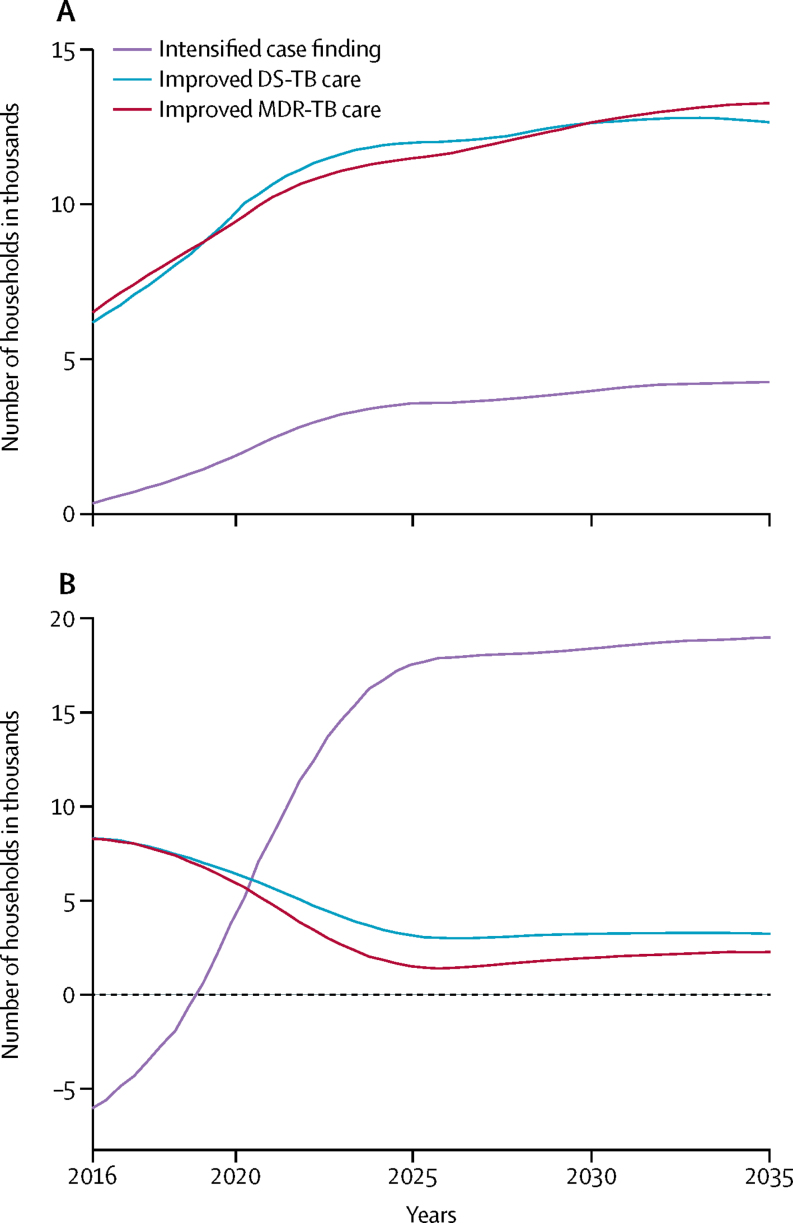
Number of households in South Africa per year with catastrophic costs averted by improved tuberculosis care and expanded access to care in 2016–35 (A) Harvard model. (B) TB Impact Model and Estimates model. DS-TB=drug-sensitive tuberculosis. MDR-TB=multidrug-resistant tuberculosis.

In South Africa, the numbers of cases of tuberculosis-related household catastrophic costs averted by improvements in care of drug-sensitive and multidrug resistant tuberculosis would be lower than in India (Figure 3, Figure 4). Expanding access to care through intensified case finding would avert about 60 000–240 000 cases of catastrophic costs in 2016–35 (table 4), which was about 5–20% of all cases incurred in the base case, although effects would not be seen for around 5–10 years (figure 4). With improvements in care of drug-sensitive tuberculosis, about 90 000–220 000 cases or 7–19% of all cases of catastrophic costs would be averted, as would 70 000–220 000 cases or 6–18% with improvements in care of multidrug-resistant tuberculosis (table 4). Households in the lowest two income quintiles would benefit the most from all three interventions. Expanded access to care would avert 65–90% of tuberculosis-related catastrophic costs in these quintiles, and improvements in care of drug-sensitive tuberculosis and multidrug-resistant tuberculosis, would each avert about 90% of cases.

In the sensitivity analysis, assigning uniform distribution of tuberculosis lessened the difference in effects between quintiles (appendix pp 10–11). With equal use of health care across quintiles, in India and South Africa, reductions remained highest in the bottom income quintile (appendix pp 12–13). Removing funeral costs due to tuberculosis-related deaths had little effect on the number of cases of catastrophic costs averted in any quintile with improvements in care of drug-sensitive and multidrug-resistant tuberculosis in both countries (appendix pp 14–15). Uniform distribution of indirect costs across quintiles led to reductions in catastrophic costs in the higher quintiles and increases in the lower quintiles (appendix pp 16–17). When we varied the threshold for estimating catastrophic costs to 10% or 40%, or used varying thresholds by quintile, the numbers of cases of catastrophic costs increased with the lower thresholds and decreased with the upper thresholds (appendix pp 18–23).

## Discussion

We developed an approach to assess the financial protection benefits of tuberculosis control in terms of cases of tuberculosis-related household catastrophic costs averted if selected interventions were implemented in India and South Africa from 2016–35. Without intervention, our base case indicated that tuberculosis-related household catastrophic costs would remain substantial in India and South Africa. Improvements in the quality of care for drug-sensitive and multidrug-resistant tuberculosis could reduce this financial burden, but even with aggressive efforts, the End TB Strategy target of no households facing tuberculosis-related catastrophic costs by 2030 would be far from being achieved in either country.

In the base case, the two lowest income quintiles bore most of the catastrophic costs, which is consistent with findings from other studies, for example in Nigeria^45^, ^46^ and Peru.^23^ We note, though, that although the gains in financial protection would be greatest in these two quintiles, the reductions would be less than 20%. Other policy actions, therefore, will be needed to reduce tuberculosis incidence and mortality. In India, the interventions included some payments to patients with tuberculosis to lessen the direct costs of accessing care, but these were not sufficiently large to combat the high indirect costs incurred. Tuberculosis is a debilitating illness that lasts long enough to adversely affect earning potential and, therefore, additional support is needed to alleviate these costs if households are to be protected. Based on a framework of tuberculosis programme activities and extensive discussions with national tuberculosis programme representatives, we studied the interventions of improved care for drug-sensitive and multidrug-resistant tuberculosis and expanded access to care through intensified case finding. Assessment of other interventions, such as expanding existing national social protection schemes for the poorest households and vulnerable populations, however, would be worthwhile, as has been proposed by WHO and partners of the newly established Health and Social Protection Action Research and Knowledge Sharing Network.^47^ The interventions we studied were focused on the delivery of core tuberculosis services, but in the future we might consider assessing systemic interventions that could reduce financial costs, such as decentralised care, fees, and transport support. Intervention coverage is only one element among many, including quality and financial architecture of health systems, that can improve care-seeking behaviour and health. The multifaceted benefits of tuberculosis interventions within the context of broader social development are intrinsically important to reductions in illness-related impoverishment.^10^, ^48^, ^49^

Our study builds on the substantial work of the TB Targets exercise,^2^, ^8^ including the use of two dynamic models of tuberculosis transmission.^35^, ^36^ Yet, our analysis had several limitations. First, we relied on modelled estimates of tuberculosis incidence and cost inputs^2^, ^8^, ^35^, ^36^ and on distributional assumptions. The lack of empirical evidence in these inputs points to the need for expanded data collection. Gathering information on tuberculosis-related costs and time losses for patients and caregivers will be very important. Tracking out-of-pocket expenditure and related catastrophic costs over time could enable testing of the effects of policies in terms of poverty reduction with the existing research platforms of randomised controlled trials or household surveys.

Second, our estimation of tuberculosis-related household catastrophic costs was rudimentary. For example, we did not consider which member of the household was affected (eg, if the main earner had tuberculosis rather than other members of the household, catastrophic costs might have been more likely) because age-stratified tuberculosis incidence was not available from the model outputs. However, how tuberculosis-related income losses by age could be differentiated without adding unnecessary complications and assumptions in the absence of empirical information is unclear. We applied the simplifying assumption of one tuberculosis case per household, whereas the number of cases might be concentrated in specific households, such as those of the poorest and most marginalised populations. Additionally, variation in indirect costs with income has a consequence on public policy valuation (ie, by placing a higher value on time losses among the higher income quintiles than those in the lower quintiles). These effects might make interventions that benefit people in the top quintiles seem to be most cost-effective, which would have implications for equitable access to care. Other elements the models might have included were financial implications related to death (eg, long-term income losses), multiple medical cost inputs (eg, because richer individuals often seek private care), discounting and income growth over time, and the presence of social-protection programmes (eg, paid sick leaves). These adjustments, though, might have led to unnecessary complexity in the findings without providing any additional insight.

Third, we represented financial protection in terms of cases of catastrophic costs averted. Other possible measures could have been cases of poverty or forced borrowing avoided. We chose household catastrophic costs averted because of its simplicity and widespread use,^44^, ^50^ but this approach has some drawbacks, such as exclusion of some individuals through the choice of threshold (eg 20% of total income).^44^ We used the sensitivity analyses to try to lessen this effect.

Fourth, while the use of two models was a strength of this study because it allowed us to consider some uncertainty in the model structure, full characterisation of uncertainty in estimates was not possible and, therefore, our findings should be interpreted with caution. Owing to the many unknowns in the intervention scenarios we assessed, we used univariate sensitivity analyses and scenario analyses to assess the effects of key factors, and did a probabilistic sensitivity analysis to determine the 95% uncertainty ranges.

Irrespective of potential limitations, we believe this study has some robust and important findings. First, substantial financial household burden is incurred because of tuberculosis in India and South Africa, with millions of households being affected. Second, improved tuberculosis control through implementation or expansion of selected services, such as those included in our models, could alleviate a notable proportion of that burden and disproportionately benefit the poorest households. Tuberculosis control alone, though, while necessary, will not be sufficient to achieve the End TB Strategy targets because the indirect costs are high. Other social-protection strategies and safety nets actions will be needed to substantially reduce catastrophic costs. In this respect, future work should estimate the additional financial resources needed to eliminate catastrophic costs by 2030, and assess whether governments should cover the full social cost of tuberculosis treatment for at-risk households in India and South Africa. This study has illustrated what financial protection might be achieved with various scenarios, but, importantly, our analysis remains only a stepping stone towards the essential process of involving policy makers and academics, who must make their own assessments of model assumptions so that our findings can best inform the policy dialogues in India and South Africa, particularly regarding feasibility, affordability, and value for money, and reflect the true scenarios of tuberculosis control in the two countries.
