# Supplementary material for: Catastrophic costs potentially averted by tuberculosis control in India and South Africa: a modelling study
Source: Lancet Glob Health. 2017 Oct 9;5(11):e1123–32. doi: 10.1016/S2214-109X(17)30341-8 (PMC5640802; doi:10.1016/S2214-109X(17)30341-8)
Supplement: Supplementary appendix [file mmc1.pdf]

THE LANCET
Global Health

Supplementary appendix

This appendix formed part of the original submission and has been peer reviewed. We post it as supplied by the authors.

Supplement to: Verguet S, Riumallo-Herl C, Gomez GB, et al. Catastrophic costs potentially averted by tuberculosis control in India and South Africa: a modelling study. *Lancet Glob Health* 2017; **5:** e1123–32.

Supplementary webappendix

Catastrophic costs averted by TB control: findings for India and South Africa from a modeling study

by

Stéphane Verguet, Carlos Riumallo-Herl, Gabriela B. Gomez, Nicolas A. Menzies, Rein M.J.G. Houben, Tom Sumner, Marek Lalli, Richard G. White, Joshua A. Salomon, Ted Cohen, Nicola Foster, Susmita Chatterjee, Sedona Sweeney, Inés Garcia Baena, Knut Lönnroth, Diana E. Weil, Anna Vassall

1. Description of the TB transmission models used.

Table S1 describes the characteristics of the two compartmental models of TB transmission used in our analysis, as developed by Menzies and colleagues (Harvard)[1] and the TB Impact Model and Estimates (TIME) modeling group.[2]

Table S1. Description of the two TB transmission models used.

Model	Type	Calibration	Age structure	Sex strata	Population strata
**Harvard**	Deterministic compartmental model	Bayesian	Single age group	No	MDR-TB, healthcare sector, TB treatment history, HIV/ART/CD4 status (9 strata)
**TIME**	Deterministic compartmental model	Manual	< 15 and 15+ years	No	MDR-TB, TB treatment history, HIV/ART/CD4 status (11 strata)

DS = drug-sensitive, MDR = multi-drug-resistant. Source: Houben and colleagues.[3]

2. Description of the TB care and prevention framework.

Figure S1 describes the patient care pathway from disease to completion of treatment (blue boxes and arrows). Areas affected for enhancing current TB programme activities (i.e. intervention scenarios) are shown in red boxes and arrows, with the number (1, 2, or 3) to link them to activities in table 1 in the main text.

Figure S1. Description of the TB care and prevention framework. Adapted from Houben and colleagues.[3]

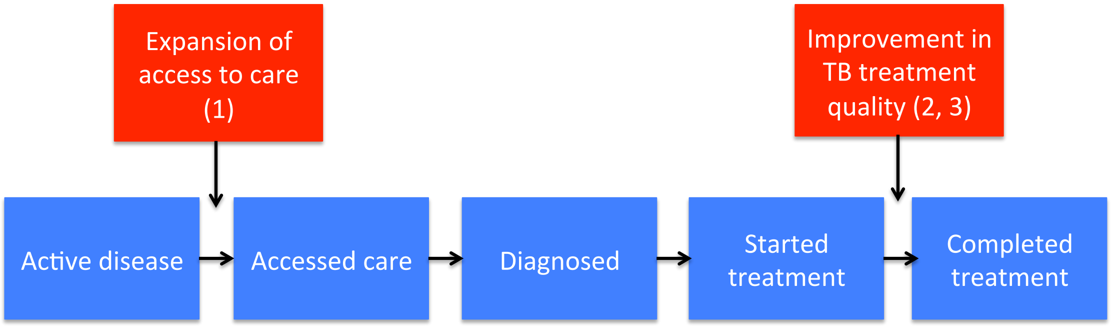

3. Estimation of patient-incurred costs

We reproduce below from Menzies and colleagues[4] the detailed inputs used in the estimation of the patient-incurred costs for both current services (i.e. base-case) and intervention services (i.e. scenarios) in India (tables S2-S3) and South Africa (tables S4-S5). We report on the patient-incurred costs as well as on the corresponding model outputs used to multiply these unit costs for patient-incurred costs.

Table S2. Unit costs for patient-incurred costs, current (base-case) services (India, US$ 2014).

Unit cost	Type	Model output	Value (US$)	Comment	Reference
**Diagnosis**					
c_DST	P_DM	Number of DST events	$0.6	Twice treatment cost visit	[5,6]
c_DST	P_DO	Number of DST events	$0.03	Twice treatment cost visit	[5,6]
c_DST	P_I	Number of DST events	$0.4	Twice treatment cost visit	[5,6]
**First line treatment**					
c_1st_line_all	P_DM	Number of first-line treatment-months	$12.2	Per month	[5,6]
c_1st_line_all	P_DO	Number of first-line treatment-months	$0.7	Per month	[5,6]
c_1st_line_all	P_I	Number of first-line treatment-months	$8.4	Per month	[5,6]
**MDR treatment**					
c_MDR_reg	P_DM	Number of MDR treatment-months	$12.2	Per month (as first-line)	[5,6]
c_MDR_reg	P_DO	Number of MDR treatment-months	$0.7	Per month (as first-line)	[4,6]
c_MDR_reg	P_I	Number of MDR treatment-months	$8.4	Per month (as first-line)	[5,6]

DST, drug sensitivity test; MDR, multi-drug-resistance; P_DM, patient costs– direct medical; P_DO, patient costs–direct other; P_I, patient costs–indirect.
Note: drug-sensitive TB care (first-line) implies six months of treatment, whereas MDR-TB care implies twenty-four months of treatment.

Table S3. Unit costs for patient-incurred costs, intervention services (intervention scenarios) (India, US$ 2014).

Unit cost	Type	Model output	Value	Comment	Reference
c_2b_DS_incentives	P_DO	Number of first-line treatment-months	-$9.2	Social support for whole treatment of US$35, plus US$20 for transport	[7]
c_2b_DS_nutrition	P_DO	Number of first-line treatment-months	-$4.2	US$25 for nutritional support cost	[7]
c_2c_MDR_incentives	P_DO	Number of MDR treatment-months	-$9.2	As with first-line treatment (per month)	[7]
c_2c_MDR_nutrition	P_DO	Number of MDR treatment-months	-$4.2	As with first-line treatment (per month)	[7]

2b–improve drug-sensitive (DS) treatment outcomes (from 75% to 85%). Patient side: incentives. Provider side: link to social welfare (including nutrition). 2c–improve multi-drug-resistant (MDR) treatment outcomes (from 48% to 67%). Patient side: incentives. Provider side: link to social welfare (including nutrition).

Note: drug-sensitive TB care (first-line) implies six months of treatment, whereas MDR-TB care implies twenty-four months of treatment.

Table S4. Unit costs for patient-incurred costs, current services (base-case) (South Africa, US$ 2014).

Unit cost	Type	Model output	Value (US$)	Comment	Reference
**First line treatment**					
c_1st_line_all	P_DM	Number of first-line treatment-months	$8.0	Per month	[8]
c_1st_line_all	P_DO	Number of first-line treatment-months	$10.2	Per month (includes loan interest)	[8]
c_1st_line_all	P_I	Number of first-line treatment-months	$20.2	Per month (reported income loss) for patient, carers and guardian	[8]
**MDR treatment**					
c_MDR_reg	P_DM	Number of MDR treatment-months	$1.2	Per month	[9]
c_MDR_reg	P_DO	Number of MDR treatment-months	$11.4	Per month (includes loan interest)	[9]
c_MDR_reg	P_I	Number of MDR treatment-months	$110.4	Per month (reported income loss) for patient, carers, and guardian	[9]

MDR, multi-drug-resistance; P_DM, patient costs–direct medical; P_DO, patient costs–direct other; P_I, patient costs– indirect.
Note: drug-sensitive TB care (first-line) implies six months of treatment, whereas MDR-TB care implies twenty-four months of treatment.

Table S5. Unit costs for patient-incurred costs, intervention services (intervention scenarios) (South Africa, US$ 2014).

Unit cost	Type	Model output	Value (US$)	Comment	Reference
c_1b_TB_symp_screen	P_DM	Per event	$0	Assumed to be zero, as part of another visit	N/A
c_1b_TB_symp_screen	P_DO	Per event	$0	Assumed to be zero, as part of another visit	N/A
c_1b_TB_symp_screen	P_I	Per event	$0.16	Mean monthly income in XTEND study group, assumed time is four minutes, plus a six-minute wait	[8,10]

P_DM, patient costs–direct medical; P_DO, patient costs–direct other; P_I, patient costs–indirect.
1b–transfer of patients from low to high quality care settings (low quality reduced from 20% to 10%).
Notes: drug-sensitive TB care (first-line) implies six months of treatment, whereas MDR-TB care implies twenty-four months of treatment.
Screen individuals who visit the health care system for TB symptoms, i.e. intensified case finding. The method will involve a person standing at a funnel point in the clinic and asking patients whether they have any TB symptoms (i.e. verbal screening). The numbers of individuals screened would ensure that 100% of unique individuals are approached by screeners, even if some then refuse or cannot give sputum. It is possible to identify unique individuals in antiretroviral therapy care, but not in general care.

4. Mathematical derivations for the estimation of TB-related catastrophic costs

This section describes the methods we used for estimating the level and distribution (across income quintiles) of the number of cases of TB-related catastrophic costs.

In each country, we divided the population into five income groups J, and we denoted:

- y, the household income;
- c_J, the costs incurred by the patient and thus his or her household.

We first allocated the number of cases of TB treated in each intervention scenario (1-3 in the main text), and given by the Harvard model[1] and the TIME model,[2] per income quintile J:

$$TxC_J = \frac{1}{5} * N * AP_J \, , \qquad (1)$$

where N was the total number of TB cases treated as given by the Harvard/TIME models, AP_J was the combined adjusted risk of TB incidence and healthcare utilization per income quintile. For example, if we denoted a_J and b_J the relative risk of TB infection across income quintiles and the relative ratio of healthcare utilization across income quintiles (table 2 in the main text), respectively, we would have: $AP_J = a_J * b_J / (\sum_{J=1}^{5} a_J * b_J)$.

TxC_J in equation (1) was estimated separately for both patients on drug-sensitive (DS)-TB care and patients on multi-drug resistance (MDR)-TB care.

Second, for each of the cases as obtained from equation (1), we assigned a household income y. Subsequently, going through each case, we counted the number of times we had

$c_J > 0.20 * y$ (patient-incurred costs exceeding 20% of total household income). That headcount gave the number of cases of household TB-related catastrophic costs incurred. Untreated TB was not considered in this estimation (equation 1 above) of the number of cases of catastrophic costs. When household costs were assigned to TB-related deaths, those TB-related deaths were allocated between those who were treated and those who were not using case fatality ratios.

5. Scenario analyses

Table S6. Estimated number of households (in 1,000s) with catastrophic costs averted over 2016-2035 in India and South Africa, when tuberculosis (TB) incidence was equal across all income quintiles, for: expansion of access to care; improvement in drug-sensitive (DS) tuberculosis (TB) care; and improvement in multi-drug resistance (MDR) TB care; as compared with the base-case. In parentheses are indicated the 95% uncertainty ranges.

a) Harvard model

India

Intervention	Total	Income quintile I	Income quintile II	Income quintile III	Income quintile IV	Income quintile V
Improvement in DS-TB care	750<br>(583 - 913)	185<br>(173 - 195)	236<br>(183 - 276)	151<br>(101 - 209)	114<br>(74 - 161)	64<br>(39 - 92)
Improvement in MDR-TB care	85<br>(61 - 109)	21<br>(18 - 22)	24<br>(16 - 32)	17<br>(9 - 28)	14<br>(6 - 23)	8<br>(3 - 15)

South Africa

Intervention	Total	Income quintile I	Income quintile II	Income quintile III	Income quintile IV	Income quintile V
Expansion of access to care	87<br>(41 - 131)	45<br>(16 - 72)	8<br>(-4 - 23)	27<br>(13 - 41)	8<br>(0 - 21)	0<br>(0 - 0)
Improvement in DS-TB care	115<br>(84 - 148)	85<br>(63 - 108)	13<br>(6 - 21)	14<br>(6 - 23)	3<br>(0 - 8)	0<br>(0 - 0)
Improvement in MDR-TB care	121<br>(91 - 154)	86<br>(65 - 107)	17<br>(10 - 25)	16<br>(8 - 25)	3<br>(0 - 8)	0<br>(0 - 0)

Note: catastrophic costs are defined as total costs exceeding 20% of total household income.
All values are in 1,000s; 95% uncertainty ranges are indicated in parentheses.

b) TIME model

India

Intervention	Total	Income quintile I	Income quintile II	Income quintile III	Income quintile IV	Income quintile V
Improvement in DS-TB care	1085<br>(864 - 1312)	156<br>(150 - 161)	275<br>(232 - 301)	300<br>(227 - 370)	223<br>(153 - 305)	131<br>(87 - 189)
Improvement in MDR-TB care	137<br>(120 - 153)	44<br>(37 - 50)	42<br>(37 - 47)	28<br>(21 - 35)	16<br>(9 - 23)	6<br>(2 - 11)

South Africa

Intervention	Total	Income quintile I	Income quintile II	Income quintile III	Income quintile IV	Income quintile V
Expansion of access to care	126<br>(95 - 158)	96<br>(71 - 121)	14<br>(4 - 24)	13<br>(5 - 21)	3<br>(-2 - 13)	0<br>(0 - 0)
Improvement in DS-TB care	52<br>(38 - 66)	38<br>(27 - 49)	8<br>(4 - 14)	4<br>(1 - 8)	2<br>(0 - 5)	0<br>(0 - 0)
Improvement in MDR-TB care	53<br>(42 - 65)	29<br>(21 - 37)	17<br>(11 - 23)	4<br>(1 - 7)	4<br>(1 - 7)	0<br>(0 - 0)

Note: catastrophic costs are defined as total costs exceeding 20% of total household income.
All values are in 1,000s; 95% uncertainty ranges are indicated in parentheses.

Table S7. Estimated number of households (in 1,000s) with catastrophic costs averted over 2016-2035 in India and South Africa when healthcare utilization was equalized across all income quintiles, for: expansion of access to care; improvement in drug-sensitive (DS) tuberculosis (TB) care; and improvement in multi-drug resistance (MDR) TB care; as compared with the base-case. In parentheses are indicated the 95% uncertainty ranges.

a) Harvard model

India

Intervention	Total	Income quintile I	Income quintile II	Income quintile III	Income quintile IV	Income quintile V
Improvement in DS-TB care	1531	939	408	130	45	10
	(1333 - 1705)	(874 - 973)	(323 - 494)	(86 - 175)	(30 - 63)	(4 - 16)
Improvement in MDR-TB care	184	115	49	14	5	1
	(158 - 209)	(106 - 121)	(36 - 63)	(7 - 23)	(1 - 9)	(0 - 3)

South Africa

Intervention	Total	Income quintile I	Income quintile II	Income quintile III	Income quintile IV	Income quintile V
Expansion of access to care	92	47	16	28	2	0
	(43 - 141)	(-3 - 96)	(-1 - 35)	(15 - 41)	(-3 - 11)	(-1 - 1)
Improvement in DS-TB care	274	226	30	15	2	0
	(226 - 321)	(190 - 261)	(19 - 43)	(7 - 23)	(0 - 7)	(0 - 1)
Improvement in MDR-TB care	268	219	30	16	3	0
	(222 - 315)	(183 - 254)	(19 - 43)	(8 - 24)	(0 - 7)	(0 - 1)

Note: catastrophic costs are defined as total costs exceeding 20% of total household income.
All values are in 1,000s; 95% uncertainty ranges are indicated in parentheses.

b) TIME model

India

Intervention	Total	Income quintile I	Income quintile II	Income quintile III	Income quintile IV	Income quintile V
Improvement in DS-TB care	1815	863	542	297	95	17
	(1652 - 1944)	(855 - 870)	(493 - 568)	(217 - 366)	(66 - 126)	(8 - 30)
Improvement in MDR-TB care	152	64	44	31	11	2
	(137 - 165)	(58 - 70)	(38 - 48)	(23 - 37)	(6 - 17)	(0 - 5)

South Africa

Intervention	Total	Income quintile I	Income quintile II	Income quintile III	Income quintile IV	Income quintile V
Expansion of access to care	226	164	25	29	9	0
	(165 - 291)	(121 - 208)	(10 - 42)	(17 - 42)	(0 - 21)	(-1 - 1)
Improvement in DS-TB care	94	68	15	8	3	0
	(73 - 117)	(52 - 85)	(8 - 22)	(3 - 14)	(0 - 7)	(0 - 1)
Improvement in MDR-TB care	68	41	17	6	4	0
	(54 - 83)	(31 - 53)	(10 - 23)	(2 - 11)	(1 - 9)	(0 - 1)

Note: catastrophic costs are defined as total costs exceeding 20% of total household income.
All values are in 1,000s; 95% uncertainty ranges are indicated in parentheses.

Table S8. Estimated number of households (in 1,000s) with catastrophic costs averted over 2016-2035 in India and South Africa, without TB-related deaths funeral costs, for: expansion of access to care; improvements in drug-sensitive (DS) tuberculosis (TB) care; and improvement in multi-drug resistance (MDR) TB care; as compared with the base-case. In parentheses are indicated the 95% uncertainty ranges.

a) Harvard model

India

Intervention	Total	Income quintile I	Income quintile II	Income quintile III	Income quintile IV	Income quintile V
Improvement in DS-TB care	1121	450	357	208	80	26
	(908 - 1311)	(429 - 464)	(265 - 433)	(136 - 276)	(52 - 115)	(13 - 41)
Improvement in MDR-TB care	120	47	42	26	9	3
	(93 - 147)	(42 - 50)	(28 - 55)	(14 - 38)	(4 - 16)	(0 - 7)

South Africa

Intervention	Total	Income quintile I	Income quintile II	Income quintile III	Income quintile IV	Income quintile V
Expansion of access to care	83	43	8	28	4	0
	(41 - 125)	(3 - 83)	(-6 - 22)	(15 - 42)	(0 - 13)	(0 - 0)
Improvement in DS-TB care	229	188	25	15	1	0
	(186 - 272)	(155 - 221)	(15 - 35)	(7 - 24)	(0 - 5)	(0 - 0)
Improvement in MDR-TB care	229	187	26	15	1	0
	(188 - 271)	(155 - 218)	(16 - 37)	(7 - 24)	(0 - 5)	(0 - 0)

Note: catastrophic costs are defined as total costs exceeding 20% of total household income.
All values are in 1,000s; 95% uncertainty ranges are indicated in parentheses.

b) TIME model

India

Intervention	Total	Income quintile I	Income quintile II	Income quintile III	Income quintile IV	Income quintile V
Improvement in DS-TB care	1449<br>(1221 - 1623)	385<br>(379 - 390)	443<br>(386 - 478)	392<br>(289 - 467)	169<br>(118 - 221)	60<br>(37 - 85)
Improvement in MDR-TB care	149<br>(135 - 163)	72<br>(67 - 76)	42<br>(35 - 47)	24<br>(17 - 30)	9<br>(4 - 14)	3<br>(0 - 6)

South Africa

Intervention	Total	Income quintile I	Income quintile II	Income quintile III	Income quintile IV	Income quintile V
Expansion of access to care	250<br>(179 - 319)	186<br>(135 - 236)	37<br>(19 - 56)	21<br>(10 - 32)	7<br>(0 - 17)	0<br>(0 - 0)
Improvement in DS-TB care	90<br>(68 - 112)	68<br>(52 - 86)	13<br>(7 - 21)	7<br>(3 - 13)	1<br>(0 - 4)	0<br>(0 - 0)
Improvement in MDR-TB care	79<br>(65 - 94)	56<br>(46 - 68)	15<br>(9 - 22)	7<br>(3 - 12)	1<br>(0 - 3)	0<br>(0 - 0)

Note: catastrophic costs are defined as total costs exceeding 20% of total household income.
All values are in 1,000s; 95% uncertainty ranges are indicated in parentheses.

Table S9. Estimated number of households (in 1,000s) with catastrophic costs averted over 2016-2035 in India and South Africa, when indirect costs are equal across income quintiles, for: expansion of access to care; improvement in drug-sensitive (DS) tuberculosis (TB) care; and improvement in multi-drug resistance (MDR) TB care; as compared with the base-case. In parentheses are indicated the 95% uncertainty ranges.

a) Harvard model

India

Intervention	Total	Income quintile I	Income quintile II	Income quintile III	Income quintile IV	Income quintile V
Improvement in DS-TB care	1060	434	345	199	62	19
	(855 - 1264)	(414 - 450)	(261 - 428)	(120 - 277)	(35 - 94)	(10 - 32)
Improvement in MDR-TB care	122	47	41	25	7	2
	(94 - 151)	(43 - 50)	(28 - 54)	(13 - 37)	(2 - 14)	(0 - 6)

South Africa

Intervention	Total	Income quintile I	Income quintile II	Income quintile III	Income quintile IV	Income quintile V
Expansion of access to care	124	68	24	27	5	0
	(76 - 173)	(28 - 108)	(1 - 55)	(13 - 41)	(0 - 13)	(0 - 0)
Improvement in DS-TB care	263	215	29	17	2	0
	(210 - 317)	(176 - 234)	(16 - 44)	(9 - 27)	(0 - 5)	(0 - 0)
Improvement in MDR-TB care	260	211	30	18	1	0
	(211 - 313)	(174 - 249)	(18 - 45)	(10 - 27)	(0 - 5)	(0 - 0)

Note: catastrophic costs are defined as total costs exceeding 20% of total household income.
All values are in 1,000s; 95% uncertainty ranges are indicated in parentheses.

b) TIME model

India

Intervention	Total	Income quintile I	Income quintile II	Income quintile III	Income quintile IV	Income quintile V
Improvement in DS-TB care	1446<br>(1226 - 1623)	378<br>(371 - 385)	474<br>(425 - 502)	377<br>(284 - 451)	173<br>(113 - 233)	44<br>(19 - 71)
Improvement in MDR-TB care	138<br>(123 - 152)	60<br>(52 - 68)	43<br>(37 - 48)	24<br>(17 - 30)	10<br>(5 - 15)	2<br>(0 - 5)

South Africa

Intervention	Total	Income quintile I	Income quintile II	Income quintile III	Income quintile IV	Income quintile V
Expansion of access to care	221<br>(171 - 272)	193<br>(152 - 232)	19<br>(6 - 34)	8<br>(1 - 17)	1<br>(-1 - 4)	0<br>(0 - 0)
Improvement in DS-TB care	98<br>(78 - 118)	82<br>(65 - 99)	10<br>(5 - 16)	5<br>(2 - 10)	0<br>(0 - 2)	0<br>(0 - 0)
Improvement in MDR-TB care	76<br>(62 - 91)	55<br>(43 - 97)	14<br>(9 - 20)	7<br>(3 - 12)	0<br>(0 - 2)	0<br>(0 - 0)

Note: catastrophic costs are defined as total costs exceeding 20% of total household income.
All values are in 1,000s; 95% uncertainty ranges are indicated in parentheses.

Table S10. Estimated number of households (in 1,000s) with catastrophic costs averted over 2016-2035 in India and South Africa, when the catastrophic costs threshold was set at 10% of total household income, for: expansion of access to care; improvement in drug-sensitive (DS) tuberculosis (TB) care; and improvement in multi-drug resistance (MDR) TB care; as compared with the base-case. In parentheses are indicated the 95% uncertainty ranges.

a) Harvard model

India

Intervention	Total	Income quintile I	Income quintile II	Income quintile III	Income quintile IV	Income quintile V
Improvement in DS-TB care	1909<br>(1704 - 2076)	434<br>(424 - 445)	580<br>(562 - 593)	528<br>(444 - 589)	275<br>(192 - 348)	91<br>(61 - 124)
Improvement in MDR-TB care	233<br>(205 - 257)	49<br>(47 - 50)	70<br>(64 - 73)	66<br>(54 - 76)	33<br>(21 - 45)	11<br>(5 - 18)

South Africa

Intervention	Total	Income quintile I	Income quintile II	Income quintile III	Income quintile IV	Income quintile V
Expansion of access to care	170<br>(105 - 236)	26<br>(-28 - 78)	93<br>(48 - 143)	37<br>(15 - 59)	4<br>(-8 - 16)	9<br>(3 - 16)
Improvement in DS-TB care	517<br>(427 - 603)	354<br>(304 - 400)	96<br>(66 - 128)	47<br>(31 - 63)	16<br>(9 - 25)	4<br>(0 - 8)
Improvement in MDR-TB care	503<br>(418 - 586)	343<br>(295 - 387)	94<br>(65 - 126)	47<br>(32 - 63)	15<br>(8 - 24)	4<br>(0 - 8)

Note: catastrophic costs are defined as total costs exceeding 10% of total household income.
All values are in 1,000s; 95% uncertainty ranges are indicated in parentheses.

b) TIME model

India

Intervention	Total	Income quintile I	Income quintile II	Income quintile III	Income quintile IV	Income quintile V
Improvement in DS-TB care	2049 (1931 - 2140)	371 (363 - 378)	517 (511 - 522)	580 (562 - 587)	380 (322 - 420)	203 (156 - 250)
Improvement in MDR-TB care	149 (135 - 162)	48 (39 - 57)	42 (37 - 47)	32 (28 - 35)	19 (14 - 22)	9 (4 - 13)

South Africa

Intervention	Total	Income quintile I	Income quintile II	Income quintile III	Income quintile IV	Income quintile V
Expansion of access to care	446 (382 - 505)	263 (230 - 296)	130 (86 - 172)	39 (24 - 56)	10 (3 - 18)	4 (0 - 9)
Improvement in DS-TB care	188 (159 - 217)	128 (111 - 145)	38 (25 - 53)	17 (10 - 24)	5 (1 - 9)	1 (0 - 3)
Improvement in MDR-TB care	116 (98 - 133)	72 (60 - 83)	27 (18 - 36)	15 (9 - 21)	3 (0 - 6)	0 (0 - 2)

Note: catastrophic costs are defined as total costs exceeding 10% of total household income.
All values are in 1,000s; 95% uncertainty ranges are indicated in parentheses.

Table S11. Estimated number of households (in 1,000s) with catastrophic costs averted over 2016-2035 in India and South Africa, when the catastrophic costs threshold was set at 40% of total household income, for: expansion of access to care; improvement in drug-sensitive (DS) tuberculosis (TB) care; and improvement in multi-drug resistance (MDR) TB care; as compared with the base-case. In parentheses are indicated the 95% uncertainty ranges.

a) Harvard model

India

Intervention	Total	Income quintile I	Income quintile II	Income quintile III	Income quintile IV	Income quintile V
Improvement in DS-TB care	439	279	104	42	10	5
	(334 - 536)	(225 - 320)	(63 - 144)	(28 - 60)	(4 - 17)	(0 - 15)
Improvement in MDR-TB care	46	28	12	5	1	1
	(31 - 62)	(19 - 36)	(5 – 2-)	(1 - 10)	(0 - 4)	(0 – 13)

South Africa

Intervention	Total	Income quintile I	Income quintile II	Income quintile III	Income quintile IV	Income quintile V
Expansion of access to care	39	36	4	-1	0	0
	(14 - 63)	(11 - 59)	(-2 - 11)	(-3 - 2)	(-1 - 1)	(0 - 0)
Improvement in DS-TB care	75	69	5	1	0	0
	(56 - 95)	(51 - 88)	(1 - 9)	(0 - 3)	(0 - 1)	(0 - 0)
Improvement in MDR-TB care	78	71	5	1	0	0
	(59 - 99)	(54 - 90)	(2 - 10)	(0 - 4)	(0 - 1)	(0 - 0)

Note: catastrophic costs are defined as total costs exceeding 40% of total household income.
All values are in 1,000s; 95% uncertainty ranges are indicated in parentheses.

b) TIME model

India

Intervention	Total	Income quintile I	Income quintile II	Income quintile III	Income quintile IV	Income quintile V
Improvement in DS-TB care	663<br>(502 - 823)	328<br>(289 - 358)	202<br>(127 - 273)	93<br>(51 - 142)	34<br>(19 - 53)	6<br>(2 - 12)
Improvement in MDR-TB care	100<br>(84 - 115)	64<br>(57 - 71)	26<br>(17 - 34)	8<br>(3 - 14)	2<br>(0 - 5)	0<br>(0 - 2)

South Africa

Intervention	Total	Income quintile I	Income quintile II	Income quintile III	Income quintile IV	Income quintile V
Expansion of access to care	76<br>(48 - 107)	71<br>(45 - 99)	4<br>(-1 - 11)	0<br>(-2 - 4)	0<br>(-1 - 1)	0<br>(0 - 0)
Improvement in DS-TB care	31<br>(20 - 42)	27<br>(17-38)	2<br>(0 - 5)	1<br>(0 - 3)	0<br>(0 - 1)	0<br>(0 - 0)
Improvement in MDR-TB care	35<br>(25 - 46)	30<br>(21 - 39)	4<br>(1 - 8)	2<br>(0 - 4)	0<br>(0 - 1)	0<br>(0 - 0)

Note: catastrophic costs are defined as total costs exceeding 40% of total household income.
All values are in 1,000s; 95% uncertainty ranges are indicated in parentheses.

Table S12. Estimated number of households (in 1,000s) with catastrophic costs averted over 2016-2035 in India and South Africa, when the catastrophic costs threshold varies by income quintile (20%, 25%, 30%, 35%, and 40% respectively) for: expansion of access to care; improvement in drug-sensitive (DS) tuberculosis (TB) care; and improvement in multi-drug resistance (MDR) TB care; as compared with the base-case. In parentheses are indicated the 95% uncertainty ranges.

a) Harvard model

India

Intervention	Total	Income quintile I	Income quintile II	Income quintile III	Income quintile IV	Income quintile V
Improvement in DS-TB care	808	430	246	118	12	2
	(669 - 940)	(403 - 449)	(174 - 318)	(75 - 159)	(6 - 21)	(0 - 8)
Improvement in MDR-TB care	91	47	29	14	1	0
	(71 - 112)	(42 - 50)	(17 - 41)	(7 - 23)	(0 - 4)	(0 - 2)

South Africa

Intervention	Total	Income quintile I	Income quintile II	Income quintile III	Income quintile IV	Income quintile V
Expansion of access to care	59	47	12	0	0	0
	(20 - 99)	(9 - 85)	(1 - 24)	(-3 - 4)	(-1 - 1)	(0 - 0)
Improvement in DS-TB care	201	184	16	1	0	0
	(163 - 240)	(148 - 219)	(9 - 25)	(0 - 4)	(0 - 1)	(0 - 0)
Improvement in MDR-TB care	200	181	17	2	0	0
	(162 - 237)	(146 - 215)	(10 - 26)	(0 - 5)	(0 - 1)	(0 - 0)

Note: all values are in 1,000s; 95% uncertainty ranges are indicated in parentheses.

b) TIME model

India

Intervention	Total	Income quintile I	Income quintile II	Income quintile III	Income quintile IV	Income quintile V
Improvement in DS-TB care	985<br>(821 - 1138)	380<br>(372 - 386)	399<br>(321 - 458)	151<br>(87 - 226)	53<br>(30 - 76)	3<br>(0 - 8)
Improvement in MDR-TB care	119<br>(106 - 133)	64<br>(57 - 70)	39<br>(32 - 46)	12<br>(6 - 19)	3<br>(0 - 7)	0<br>(0 - 1)

South Africa

Intervention	Total	Income quintile I	Income quintile II	Income quintile III	Income quintile IV	Income quintile V
Expansion of access to care	198<br>(161 - 237)	164<br>(133 - 197)	30<br>(18 - 44)	3<br>(-2 - 13)	0<br>(-1 - 1)	0<br>(0 - 0)
Improvement in DS-TB care	79<br>(62 - 97)	68<br>(53 - 83)	10<br>(4 - 16)	2<br>(0 - 5)	0<br>(0 - 1)	0<br>(0 - 0)
Improvement in MDR-TB care	63<br>(49 - 76)	49<br>(38 - 60)	11<br>(6 - 17)	2<br>(0 - 5)	0<br>(0 - 1)	0<br>(0 - 0)

Note: all values are in 1,000s; 95% uncertainty ranges are indicated in parentheses.

6. Univariate sensitivity analyses

Table S13. Estimated number of households (in 1,000s) with catastrophic costs averted over 2016-2035 in India and South Africa, with 50% lower direct costs, for: expansion of access to care; improvement in drug sensitive (DS) tuberculosis (TB) care; and improvement in multi-drug resistance (MDR) TB care; as compared with the base-case. In parentheses are indicated the 95% uncertainty ranges.

a) Harvard model

India

Intervention	Total	Income quintile I	Income quintile II	Income quintile III	Income quintile IV	Income quintile V
Improvement in DS-TB care	398<br>(306 - 507)	265<br>(222 - 311)	79<br>(48 - 116)	36<br>(18 - 63)	15<br>(7 - 24)	3<br>(0 - 8)
Improvement in MDR-TB care	41<br>(27 - 57)	27<br>(19 - 35)	8<br>(3 - 15)	4<br>(0 - 10)	2<br>(0 - 5)	0<br>(0 - 2)

South Africa

Intervention	Total	Income quintile I	Income quintile II	Income quintile III	Income quintile IV	Income quintile V
Expansion of access to care	21<br>(-12 - 55)	5<br>(-26 - 36)	16<br>(5 - 27)	1<br>(-3 - 7)	0<br>(-1 - 1)	0<br>(0 - 0)
Improvement in DS-TB care	143<br>(116 - 173)	129<br>(104 - 155)	12<br>(6 - 20)	2<br>(0 - 5)	0<br>(0 - 1)	0<br>(0 - 0)
Improvement in MDR-TB care	142<br>(115 - 171)	127<br>(103 - 153)	13<br>(6 - 20)	2<br>(0 - 6)	0<br>(0 - 1)	0<br>(0 - 0)

Note: catastrophic costs are defined as total costs exceeding 20% of total household income.
All values are in 1,000s; 95% uncertainty ranges are indicated in parentheses.

b) TIME model

India

Intervention	Total	Income quintile I	Income quintile II	Income quintile III	Income quintile IV	Income quintile V
Improvement in DS-TB care	628<br>(480 - 775)	313<br>(268 - 348)	218<br>(156 - 273)	62<br>(28 - 109)	31<br>(16 - 49)	5<br>(1 - 10)
Improvement in MDR-TB care	92<br>(77 - 108)	57<br>(49 - 65)	26<br>(18 - 34)	7<br>(2 - 12)	2<br>(0 - 5)	0<br>(0 - 1)

South Africa

Intervention	Total	Income quintile I	Income quintile II	Income quintile III	Income quintile IV	Income quintile V
Expansion of access to care	130<br>(97 - 162)	112<br>(85 - 139)	16<br>(6 - 27)	2<br>(-2 - 8)	0<br>(-1 - 1)	0<br>(0 - 0)
Improvement in DS-TB care	47<br>(33 - 61)	40<br>(28 - 53)	5<br>(1 - 10)	2<br>(0 - 4)	0<br>(0 - 1)	0<br>(0 - 0)
Improvement in MDR-TB care	43<br>(31 - 55)	33<br>(24 - 44)	7<br>(2 - 12)	2<br>(0 - 5)	0<br>(0 - 1)	0<br>(0 - 0)

Note: catastrophic costs are defined as total costs exceeding 20% of total household income.
All values are in 1,000s; 95% uncertainty ranges are indicated in parentheses.

Table S14. Estimated number of households (in 1,000s) with catastrophic costs averted over 2016-2035 in India and South Africa, with 50% higher direct costs, for: expansion of access to care; improvement in drug sensitive (DS) tuberculosis (TB) care; and improvement in multi-drug resistance (MDR) TB care; as compared with the base-case. In parentheses are indicated the 95% uncertainty ranges.

a) Harvard model

India

Intervention	Total	Income quintile I	Income quintile II	Income quintile III	Income quintile IV	Income quintile V
Improvement in DS-TB care	1546<br>(1303 - 1753)	452<br>(441 - 462)	504<br>(435 - 553)	402<br>(299 - 493)	145<br>(92 - 199)	43<br>(22 - 66)
Improvement in MDR-TB care	183<br>(150 - 212)	49<br>(47 - 51)	61<br>(50 - 69)	51<br>(35 - 65)	17<br>(9 - 27)	5<br>(1 - 10)

South Africa

Intervention	Total	Income quintile I	Income quintile II	Income quintile III	Income quintile IV	Income quintile V
Expansion of access to care	82<br>(33 - 133)	35<br>(-6 - 76)	12<br>(-9 - 37)	7<br>(-9 - 25)	28<br>(15 - 40)	0<br>(0 - 2)
Improvement in DS-TB care	347<br>(287 - 407)	256<br>(212 - 299)	53<br>(38 - 71)	26<br>(17 - 36)	12<br>(5 - 20)	0<br>(0 - 1)
Improvement in MDR-TB care	342<br>(284 - 402)	250<br>(207 - 292)	54<br>(39 - 71)	27<br>(18 - 37)	11<br>(4 - 19)	0<br>(0 - 1)

Note: catastrophic costs are defined as total costs exceeding 20% of total household income.
All values are in 1,000s; 95% uncertainty ranges are indicated in parentheses.

b) TIME model

India

Intervention	Total	Income quintile I	Income quintile II	Income quintile III	Income quintile IV	Income quintile V
Improvement in DS-TB care	1841 (1682 - 1982)	378 (371 - 385)	516 (507 - 522)	529 (474 - 571)	294 (224 - 354)	124 (87 - 165)
Improvement in MDR-TB care	156 (143 - 168)	61 (53 - 68)	45 (40 - 49)	30 (25 - 34)	15 (10 - 20)	5 (2 - 9)

South Africa

Intervention	Total	Income quintile I	Income quintile II	Income quintile III	Income quintile IV	Income quintile V
Expansion of access to care	300 (213 - 381)	210 (152 - 263)	54 (25 - 83)	22 (10 - 35)	14 (4 - 25)	0 (0 - 0)
Improvement in DS-TB care	119 (92 - 145)	84 (64 - 103)	19 (11 - 29)	13 (7 - 20)	3 (0 - 6)	0 (0 - 0)
Improvement in MDR-TB care	91 (75 - 107)	57 (45 - 69)	20 (14 - 27)	12 (7 - 18)	1 (0 - 4)	0 (0 - 0)

Note: catastrophic costs are defined as total costs exceeding 20% of total household income.
All values are in 1,000s; 95% uncertainty ranges are indicated in parentheses.

Table S15. Estimated number of households (in 1,000s) with catastrophic costs averted over 2016-2035 in India and South Africa, with 50% lower funeral costs, for: expansion of access to care; improvement in drug sensitive (DS) tuberculosis (TB) care; and improvement in multi-drug resistance (MDR) TB care; as compared with the base-case. In parentheses are indicated the 95% uncertainty ranges.

a) Harvard model

India

Intervention	Total	Income quintile I	Income quintile II	Income quintile III	Income quintile IV	Income quintile V
Improvement in DS-TB care	1087<br>(888 - 1294)	423<br>(392 - 447)	349<br>(265 - 433)	212<br>(152 - 278)	80<br>(54 - 112)	23<br>(10 - 37)
Improvement in MDR-TB care	124<br>(96 - 152)	45<br>(39 - 49)	41<br>(28 - 54)	26<br>(16 - 38)	9<br>(4 - 16)	3<br>(0 - 6)

South Africa

	Total	Income quintile I	Income quintile II	Income quintile III	Income quintile IV	Income quintile V
Expansion of access to care	88<br>(45 - 132)	45<br>(10 - 79)	16<br>(0 - 35)	22<br>(10 - 34)	5<br>(-1 - 16)	0<br>(0 - 0)
Improvement in DS-TB care	198<br>(157 - 242)	164<br>(132 - 197)	19<br>(10 - 29)	14<br>(6 - 22)	2<br>(0 - 6)	0<br>(0 - 0)
Improvement in MDR-TB care	199<br>(159 - 242)	163<br>(132 - 196)	20<br>(11 - 30)	14<br>(7 - 22)	2<br>(0 - 6)	0<br>(0 - 0)

Note: catastrophic costs are defined as total costs exceeding 20% of total household income.
All values are in 1,000s; 95% uncertainty ranges are indicated in parentheses.

b) TIME model

India

Intervention	Total	Income quintile I	Income quintile II	Income quintile III	Income quintile IV	Income quintile V
Improvement in DS-TB care	1477<br>(1267 - 1660)	383<br>(376 - 388)	465<br>(406 - 501)	394<br>(305 - 470)	176<br>(126 - 236)	59<br>(37 - 82)
Improvement in MDR-TB care	148<br>(133 - 161)	68<br>(62 - 73)	43<br>(38 - 48)	24<br>(18 - 31)	10<br>(5 - 15)	2<br>(0 - 6)

South Africa

Intervention	Total	Income quintile I	Income quintile II	Income quintile III	Income quintile IV	Income quintile V
Expansion of access to care	205<br>(158 - 262)	153<br>(117 - 196)	22<br>(9 - 37)	26<br>(15 - 39)	4<br>(-1 - 11)	0<br>(0 - 0)
Improvement in DS-TB care	86<br>(67 - 107)	67<br>(51 - 83)	11<br>(5 - 17)	8<br>(3 - 14)	1<br>(0 - 3)	0<br>(0 - 0)
Improvement in MDR-TB care	75<br>(62 - 89)	53<br>(42 - 64)	15<br>(9 - 21)	7<br>(3 - 12)	0<br>(0 - 2)	0<br>(0 - 0)

Note: catastrophic costs are defined as total costs exceeding 20% of total household income.
All values are in 1,000s; 95% uncertainty ranges are indicated in parentheses.

Table S16. Estimated number of households (in 1,000s) with catastrophic costs averted over 2016-2035 in India and South Africa, with 50% higher funeral costs, for: expansion of access to care; improvement in drug sensitive (DS) tuberculosis (TB) care; and improvement in multi-drug resistance (MDR) TB care; as compared with the base-case. In parentheses are indicated the 95% uncertainty ranges.

a) Harvard model

India

Intervention	Total	Income quintile I	Income quintile II	Income quintile III	Income quintile IV	Income quintile V
Improvement in DS-TB care	1015<br>(825 - 1216)	404<br>(371 - 431)	345<br>(264 - 419)	164<br>(108 - 233)	71<br>(44 - 107)	30<br>(19 - 44)
Improvement in MDR-TB care	117<br>(91 - 146)	45<br>(39 - 49)	41<br>(28 - 53)	20<br>(11 - 32)	8<br>(3 - 15)	4<br>(0 - 8)

South Africa

Intervention	Total	Income quintile I	Income quintile II	Income quintile III	Income quintile IV	Income quintile V
Expansion of access to care	112<br>(58 - 165)	69<br>(27 - 111)	18<br>(4 - 34)	18<br>(7 - 29)	8<br>(0 - 19)	0<br>(0 - 0)
Improvement in DS-TB care	238<br>(188 - 290)	201<br>(161 - 242)	22<br>(13 - 33)	12<br>(5 - 20)	3<br>(0 - 7)	0<br>(0 - 0)
Improvement in MDR-TB care	236<br>(187 - 285)	197<br>(157 - 236)	23<br>(14 - 34)	13<br>(6 - 21)	3<br>(0 - 7)	0<br>(0 - 0)

Note: catastrophic costs are defined as total costs exceeding 20% of total household income.
All values are in 1,000s; 95% uncertainty ranges are indicated in parentheses.

b) TIME model

India

Intervention	Total	Income quintile I	Income quintile II	Income quintile III	Income quintile IV	Income quintile V
Improvement in DS-TB care	1472<br>(1244 - 1648)	374<br>(365 - 381)	472<br>(422 - 502)	396<br>(313 - 467)	180<br>(105 - 242)	50<br>(24 - 74)
Improvement in MDR-TB care	133<br>(117 - 149)	55<br>(46 - 64)	42<br>(35 - 47)	24<br>(18 - 30)	10<br>(4 - 15)	2<br>(0 - 5)

South Africa

Intervention	Total	Income quintile I	Income quintile II	Income quintile III	Income quintile IV	Income quintile V
Expansion of access to care	186<br>(150 - 222)	146<br>(118 - 173)	18<br>(7 - 30)	19<br>(6 - 33)	4<br>(-1 - 12)	0<br>(0 - 0)
Improvement in DS-TB care	85<br>(68 - 103)	67<br>(53 - 81)	11<br>(5 - 17)	6<br>(2 - 12)	1<br>(0 - 3)	0<br>(0 - 0)
Improvement in MDR-TB care	68<br>(54 - 82)	47<br>(36 - 58)	13<br>(8 - 19)	7<br>(3 - 12)	1<br>(0 - 2)	0<br>(0 - 0)

Note: catastrophic costs are defined as total costs exceeding 20% of total household income.
All values are in 1,000s; 95% uncertainty ranges are indicated in parentheses.

Table S17. Estimated number of households (in 1,000s) with catastrophic costs averted over 2016-2035 in India and South Africa, with 50% lower utilization rates, for: expansion of access to care; improvement in drug sensitive (DS) tuberculosis (TB) care; and improvement in multi-drug resistance (MDR) TB care; as compared with the base-case. In parentheses are indicated the 95% uncertainty ranges.

a) Harvard model

India

Intervention	Total	Income quintile I	Income quintile II	Income quintile III	Income quintile IV	Income quintile V
Improvement in DS-TB care	1414<br>(1214 - 1608)	824<br>(777 - 857)	395<br>(299 - 475)	146<br>(95 - 208)	34<br>(19 - 55)	16<br>(8 - 25)
Improvement in MDR-TB care	169<br>(142 - 196)	101<br>(93 - 107)	46<br>(32 - 59)	16<br>(8 - 26)	4<br>(1 - 9)	2<br>(0 - 5)

South Africa

Intervention	Total	Income quintile I	Income quintile II	Income quintile III	Income quintile IV	Income quintile V
Expansion of access to care	75<br>(30 - 122)	43<br>(8 - 79)	4<br>(-11 - 20)	27<br>(10 - 42)	1<br>(-3 - 9)	0<br>(-1 - 0)
Improvement in DS-TB care	217<br>(173 - 263)	176<br>(142 - 210)	26<br>(17 - 38)	13<br>(5 - 22)	2<br>(0 - 5)	0<br>(0 - 0)
Improvement in MDR-TB care	214<br>(171 - 259)	171<br>(138 - 205)	26<br>(17 - 38)	14<br>(6 - 23)	2<br>(0 - 5)	0<br>(0 - 0)

Note: catastrophic costs are defined as total costs exceeding 20% of total household income.
All values are in 1,000s; 95% uncertainty ranges are indicated in parentheses.

b) TIME model

India

Intervention	Total	Income quintile I	Income quintile II	Income quintile III	Income quintile IV	Income quintile V
Improvement in DS-TB care	1691<br>(1513 - 1837)	756<br>(745 - 764)	507<br>(455 - 540)	296<br>(219 - 362)	100<br>(64 - 140)	32<br>(17 - 47)
Improvement in MDR-TB care	146<br>(131 - 160)	61<br>(55 - 67)	46<br>(40 - 51)	26<br>(19 - 32)	11<br>(6 - 16)	2<br>(0 - 5)

South Africa

Intervention	Total	Income quintile I	Income quintile II	Income quintile III	Income quintile IV	Income quintile V
Expansion of access to care	237<br>(192 - 286)	191<br>(161 - 223)	28<br>(10 - 52)	12<br>(3 - 22)	6<br>(-1 - 16)	0<br>(-1 - 1)
Improvement in DS-TB care	87<br>(68 - 107)	70<br>(54 - 86)	10<br>(4 - 17)	5<br>(1 - 9)	2<br>(0 - 6)	0<br>(0 - 1)
Improvement in MDR-TB care	75<br>(61 - 90)	47<br>(36 - 58)	18<br>(13 - 25)	7<br>(3 - 12)	2<br>(0 - 5)	0<br>(0 - 1)

Note: catastrophic costs are defined as total costs exceeding 20% of total household income.
All values are in 1,000s; 95% uncertainty ranges are indicated in parentheses.

Table S18. Estimated number of households (in 1,000s) with catastrophic costs averted over 2016-2035 in India and South Africa, with 50% higher utilization rates, for: expansion of access to care; improvement in drug sensitive (DS) tuberculosis (TB) care; and improvement in multi-drug resistance (MDR) TB care; as compared with the base-case. In parentheses are indicated the 95% uncertainty ranges.

a) Harvard model

India

Intervention	Total	Income quintile I	Income quintile II	Income quintile III	Income quintile IV	Income quintile V
Improvement in DS-TB care	1401<br>(1215 - 1577)	808<br>(759 - 835)	377<br>(291 - 456)	153<br>(107 - 208)	47<br>(31 - 67)	15<br>(7 - 25)
Improvement in MDR-TB care	170<br>(145 - 194)	104<br>(96 - 109)	45<br>(31 - 57)	16<br>(8 - 26)	4<br>(1 - 9)	1<br>(0 - 4)

South Africa

Intervention	Total	Income quintile I	Income quintile II	Income quintile III	Income quintile IV	Income quintile V
Expansion of access to care	95<br>(50 - 142)	72<br>(34 - 112)	2<br>(-11 - 16)	17<br>(6 - 29)	4<br>(-3 - 13)	0<br>(-2 - 1)
Improvement in DS-TB care	183<br>(142 - 227)	144<br>(111 - 181)	24<br>(15 - 33)	10<br>(4 - 17)	5<br>(1 - 9)	0<br>(0 - 2)
Improvement in MDR-TB care	186<br>(146 - 229)	139<br>(106 - 174)	28<br>(18 - 38)	13<br>(6 - 21)	6<br>(1 - 11)	1<br>(0 - 3)

b) TIME model

India

Intervention	Total	Income quintile I	Income quintile II	Income quintile III	Income quintile IV	Income quintile V
Improvement in DS-TB care	1401<br>(1215 - 1577)	808<br>(759 - 835)	377<br>(291 - 456)	153<br>(107 - 208)	47<br>(31 - 67)	15<br>(7 - 25)
Improvement in MDR-TB care	170<br>(145 - 131)	104<br>(96 - 109)	45<br>(31 - 57)	16<br>(8 - 26)	4<br>(1 - 9)	1<br>(0 - 4)

South Africa

Intervention	Total	Income quintile I	Income quintile II	Income quintile III	Income quintile IV	Income quintile V
Expansion of access to care	95<br>(50 - 142)	72<br>(34 - 112)	2<br>(-11 - 16)	17<br>(6 - 29)	4<br>(-3 - 13)	0<br>(-2 - 1)
Improvement in DS-TB care	183<br>(142 - 227)	144<br>(111 - 181)	24<br>(15 - 33)	10<br>(4 - 17)	5<br>(1 - 9)	0<br>(0 - 2)
Improvement in MDR-TB care	186<br>(146 - 229)	139<br>(106 - 174)	28<br>(18 - 38)	13<br>(6 - 21)	6<br>(1 - 11)	1<br>(0 - 3)

Note: catastrophic costs are defined as total costs exceeding 20% of total household income.
All values are in 1,000s; 95% uncertainty ranges are indicated in parentheses.

References

1. Menzies NA, Cohen T, Lin H-H, et al., Population health impact and cost-effectiveness of tuberculosis diagnosis with Xpert MTB/RIF: a dynamic simulation and economic evaluation. PLoS Medicine 2012; 9(11):e1001347.
2. Houben RMGJ, Lalli M, Sumner T, et al. TIME Impact - a new user-friendly TB model to inform TB policy decisions. BMC Medicine 2016;14(1):1-10.
3. Houben RMGJ, Menzies NA, Sumner T, et al. Feasibility of achieving the 2025 WHO Global TB Targets in South Africa, China and India: a combined analysis of 11 models. Lancet Global Health 2016; 4:e806-815.
4. Menzies NA, Gomez GB, Bozzani F, et al. Cost-effectiveness and resource implications of aggressive TB control in China, India and South Africa: a combined analysis of nine models. Lancet Global Health 2016; 4:e816-826.
5. Pantoja A, Floyd K, Unnikrishnan KP, et al. Economic evaluation of public-private mix for tuberculosis care and control, India. Part I. Socio-economic profile and costs among tuberculosis patients. International Journal of Tuberculosis and Lung Disease 2009; 13(6):698-704.
6. John KR, Daley P, Kincler N, Oxlade O, Menzies D. Costs incurred by patients with pulmonary tuberculosis in rural India. International Journal of Tuberculosis and Lung Disease 2009; 13(10):1281-7.
7. National Tuberculosis Programme. Personal Communication.
8. Foster N, Vassall A, Cleary S, et al. The economic burden of TB diagnosis and treatment in South Africa. Social Science and Medicine 2015; 130:e27.
9. Ramma L, Cox H, Wilkinson L, et al. Patients' costs associated with seeking and accessing treatment for drug-resistant tuberculosis in South Africa. International Journal of Tuberculosis and Lung Disease 2015; 19(12):1513-9.
10. Hausler HP, Sinanovic E, Kumaranayake L, et al. Costs of measures to control tuberculosis/HIV in public primary care facilities in Cape Town, South Africa. Bulletin of the World Health Organization 2006; 84(7):528-36.
